# Pericapsular Nerve Group (PENG) Block in Combination With the Quadratus Lumborum Block Analgesia for Revision Total Hip Arthroplasty: A Retrospective Case Series

**DOI:** 10.7759/cureus.12233

**Published:** 2020-12-23

**Authors:** Promil Kukreja, Braden Schuster, Theresa Northern, Sandra Sipe, Sameer Naranje, Hari Kalagara

**Affiliations:** 1 Anesthesiology and Perioperative Medicine, University of Alabama at Birmingham, Birmingham, USA; 2 Orthopaedic Surgery, University of Alabama at Birmingham, Birmingham, USA

**Keywords:** total hip arthroplasty, pericapsular nerve group (peng), quadratus lumborum, analgesia, regional anesthesia, nerve block

## Abstract

Orthopedic procedures involving the hip have remained challenging for regional anesthesia given the complex innervation, painful nature contributing to difficulty positioning, and a desire to maintain mobility to hasten postoperative recovery. The revision total hip arthroplasty (THA) poses a greater challenge for an effective regional analgesia due to complex surgical approach, scarring from previous surgery and limited patient mobility. The quadratus lumborum (QL) block has demonstrated to provide effective analgesia for primary hip surgery in recent studies. The pericapsular nerve group (PENG) block has also shown to provide analgesia in patients with hip fractures. There is no standard of care regional anesthesia technique for hip surgeries, and the regional practice varies widely among anesthesia providers. This retrospective case series studied the effect of combining the QL with PENG block on the revision THA analgesia.

## Introduction

Given the prevalence of osteoarthritis amongst the increasingly aging population and subsequent need for total hip arthroplasty, the frequency of revision hip surgeries is expected to rise. It is estimated that more than 2.5 million patients in the United States alone have had a total hip arthroplasty (THA) [1]. Revisions become necessary for a variety of reasons: failed implant whether from wear or loosening, infections, recall of prosthesis. Analgesia for THA is difficult even for primary cases, given the complex innervation of hip anatomy, but the challenges are compounded in revision surgeries given the potential for scarred anatomical landmarks and potentially higher level of pain. Painful positioning sometimes also limits the block options when choosing the appropriate block. Controlling acute post-surgical pain is especially important because of its potential risk factor for future chronic pain, as the intensity of early postoperative pain seems to be more of an indicator to chronic pain than preoperative pain levels [2]. The advantage of superior analgesia is sometimes competing against the desire to maintain mobility to limit the length of stay in the hospital. No single method has proven efficacious, with many options being explored. There is no universal consensus about the optimal analgesic intervention for primary or revision total hip arthroplasty. The importance of multimodal anesthesia that seeks to limit the use of opioids with regional anesthesia as the cornerstone is important because of the significant side effects of opioids, especially in the geriatric population where limiting delirium, sedation, nausea and vomiting and need for other anti-emetic medications, is paramount. Goals of care include early mobilization, improved postoperative outcomes and reducing hospital length of stay [3-6].

The hip has both an anterior and posterior capsule, but the anterior capsule contains predominantly nociceptive fibers while the posterior predominantly has mechanoreceptors. Favoring analgesia without limiting mobility, the anterior capsule is an ideal target for regional anesthesia techniques. In addition, the hip joint is innervated by both the lumbar (L1-4) and sacral (L4-S4) plexus [7]. The pericapsular nerve group (PENG) block, a novel technique first described in 2018, is an interfascial plane block that targets the articular branches of the femoral, obturator, and accessory obturator nerves at the hip [8]. The block is performed by easily identifiable bony landmarks, the anterior inferior iliac spine and the iliopubic eminence, as the articular branches of the femoral, obturator and accessory obturator nerve are consistently found here. A distinct advantage of the PENG block is supine positioning, especially important in chronic pain patients or those with an acute hip fracture. In addition, because it only targets the sensory articular branches, it has been associated with no significant motor weakness. A disadvantage is that it cannot be used as a sole anesthetic block. In this retrospective case series, we combined the PENG block with the QL block to study their effect on post-operative analgesia and opioid consumption.

Quadratus lumborum (QL) block has proven effective analgesia for total hip arthroplasty and decreasing opioid requirements for up to 48 hours postoperatively, due potentially to its spread to the paravertebral space [9]. The proposed mechanism of action of QL is guided by the anatomy, based on the close proximity of anterior border of QL muscle to lumbar plexus and paravertebral space. Though interfascial blocks are known to have considerable variability, it has consistently been reported to achieve a larger dermatomal distribution between T7-L2. Potentially limiting its clinical use is the deep location of the quadratus lumborum muscle and its close relationship to abdominal and retroperitoneal viscera; therefore, it requires a high level of vigilance and advanced technical competency. In addition, the bleeding risk is compounded by the proximity of the abdominal branches of the lumbar arteries course [10]. Another drawback is the potential, albeit small risk, of local anesthetic spread to the lumbar plexus leading to prolonged motor weakness, delaying mobilization and ultimately potentially discharge [11]. Compared with lateral QL block, posterior and anterior QL block was associated with a high incidence of quadriceps muscle weakness [12]. Because of the large volume of local anesthetic inherently necessary in fascial plane blocks, local anesthetic systemic toxicity is always a potential concern.

We hypothesized that the combination of PENG with QL block would provide adequate analgesia for the revision THAs, while also limiting motor weakness and thus improving postoperative outcomes. We examined whether addition of pericapsular nerve group (PENG) block with the quadratus lumborum block would provide superior analgesia and would prove to decrease opioid requirements and pain scores.

## Materials and methods

This retrospective case series included patients undergoing revision total hip arthroplasty (THA) at a tertiary academic medical center. Sixteen patients undergoing revision THA provided written consent for the nerve block(s). Eight patients had a QL and PENG block performed and eight patients had only a QL block performed. The nerve block(s) were performed preoperatively with the patient in a supine position for the PENG block and in lateral decubitus position for the QL block. Seven out of eight revision THA patients in the PENG and QL group were done under general endotracheal tube anesthesia (GETA) with a lone patient done under spinal anesthesia. All eight revision THA patients in the QL only group were performed under GETA. The visual analog scores of pain in the post-anesthesia care unit (PACU) and at 6, 12, and 24 hours after surgery were obtained. In addition, cumulative oral morphine equivalent (OME) usage was obtained for PACU, for the first 6 hours, 6-12 hours, and 12-24 hours postoperatively.

Ultrasound-guided technique for PENG block

**Figure 1:**
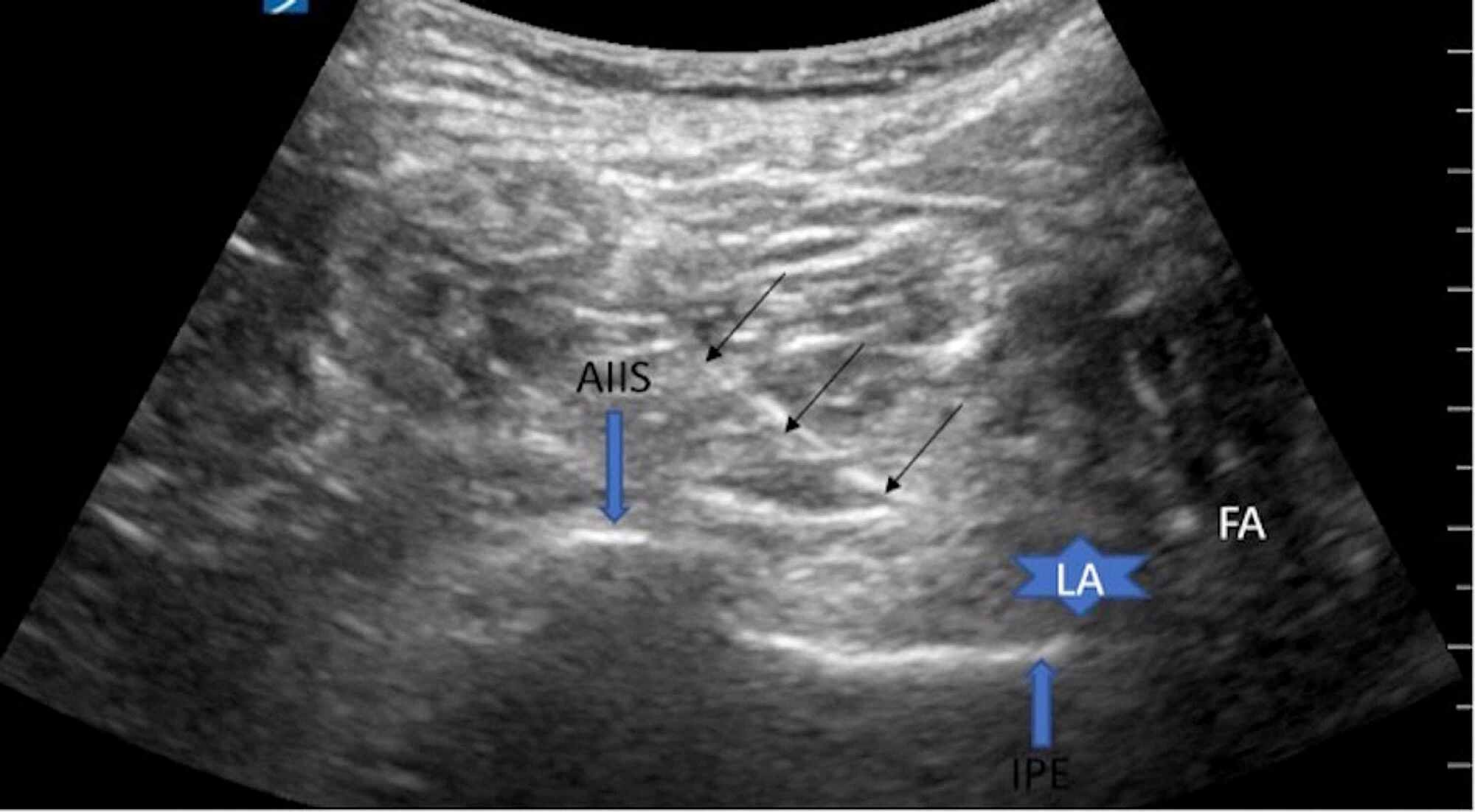
PENG BLOCK: Three black arrows indicate needle traversing from lateral to medial passing the anterior inferior iliac spine (AIIS) and depositing local anesthetic (LA) along iliopubic eminence (IPE) just lateral to femoral artery (FA).

A low-frequency curvilinear transducer was placed in the transverse plane over the anterior inferior iliac spine (AIIS) and moved over inferiorly to visualize the pubic ramus. The femoral artery and iliopubic eminence (IPE) were then visualized (Figure 1). Using in-plane technique 10 cm echogenic 21 gauge needle was advanced from lateral to medial direction, and 20 ml of local anesthetic 0.5% ropivacaine was deposited between the psoas tendon anteriorly and pubic ramus posteriorly (Figure 1).

Ultrasound-guided technique for transmuscular (anterior) QL block

**Figure 2:**
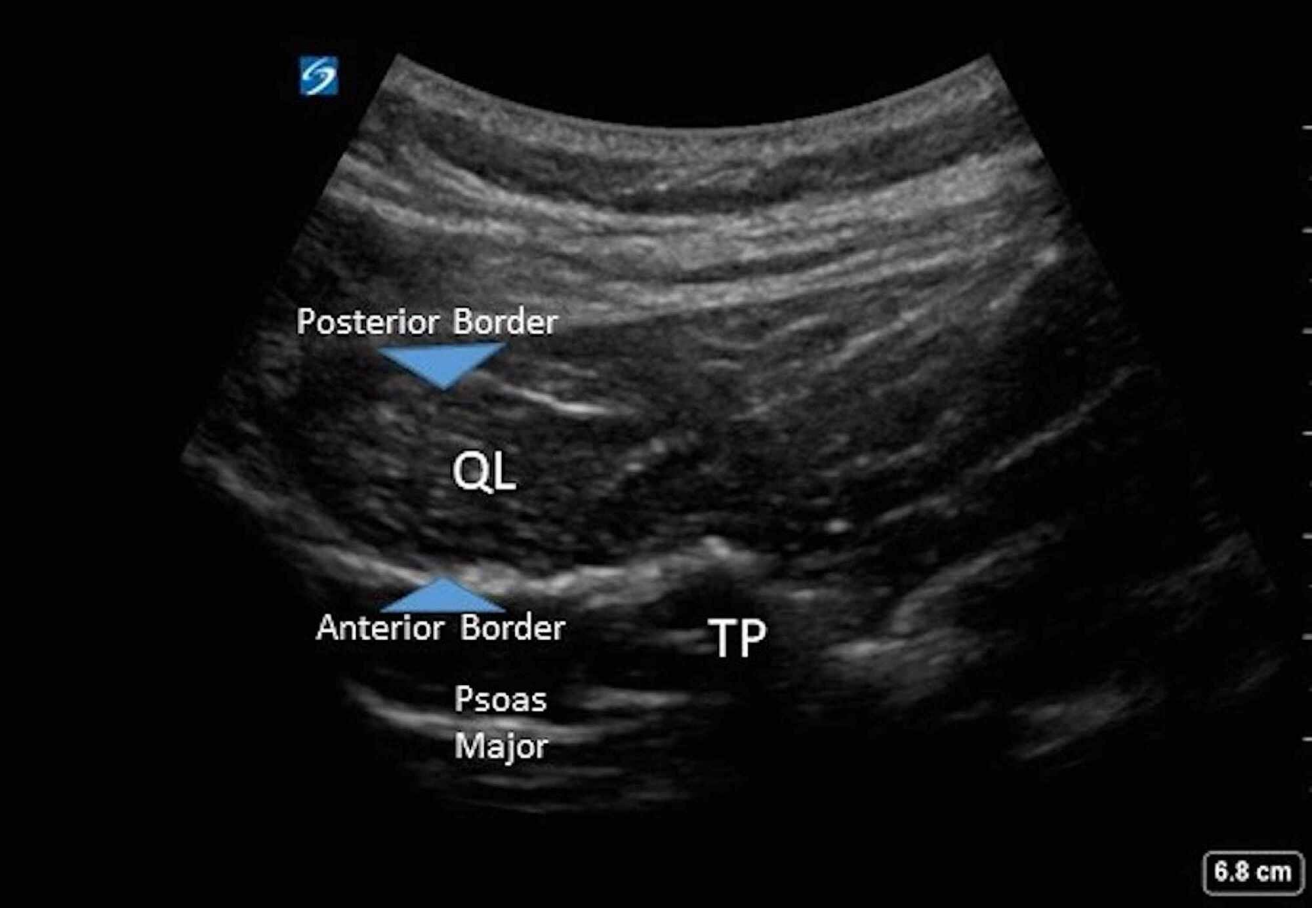
Quadratus lumborum (QL) block anatomy: In anterior QL block, local anesthetic is injected along anterior border of QL muscle in fascial plane between QL and psoas major muscles.

A low-frequency curvilinear transducer was placed in the axial plane over the spinous process at the level of L4. The probe was moved laterally until the transverse process of L4 was visualized. The “shamrock sign” was then visualized which consists of the following: QL muscle laterally, erector spinae muscle posteriorly, psoas major muscle anteriorly, and transverse process of L4 medially (stem of leaf). Using in-plane technique either a 10 cm echogenic 21g short bevel needle (seven out of 16 patients) or 9 cm 18g Tuohy needle (nine out of 16 patients) was advanced from a posterior (lateral) to anterior (medial) direction and 25 ml of local anesthetic 0.25% Bupivacaine was deposited in the fascial plane between the QL and psoas major muscles (Figure 2).

## Results

In the PENG plus QL group, average pain scores across all recorded time points during the study period were lower when compared to the QL only group (Table 1) with statistically significant p values (<0.05) at the 6 and 24 hour time point. The pain scores were not normally distributed and a non-parametric analysis using Mann-Whitney U test was performed (SPSS v.22, IBM Corp., Armonk, NY). In the QL only group, average pain scores peaked at 6.0 while in the PENG plus QL group the average pain score peaked at 3.5. There was no statistically significant difference between the ages of the two groups (Table 1).

**Table 1:** Post-operative pain scores of revision total hip arthroplasty patients after quadratus lumborum (QL) block versus pericapsular nerve group (PENG) block plus QL block.

	QL N = 8	PENG + QL N = 8	P-value
Age (years)	59.6	59.8	0.983
Pain Scores			
PACU	6.0	3.5	0.218
0 to < 6 hrs	5.6	2.1	0.037
6 to < 12 hrs	3.8	2.3	0.418
12 to 24 hrs	4.8	1.6	0.005

**Figure 3:**
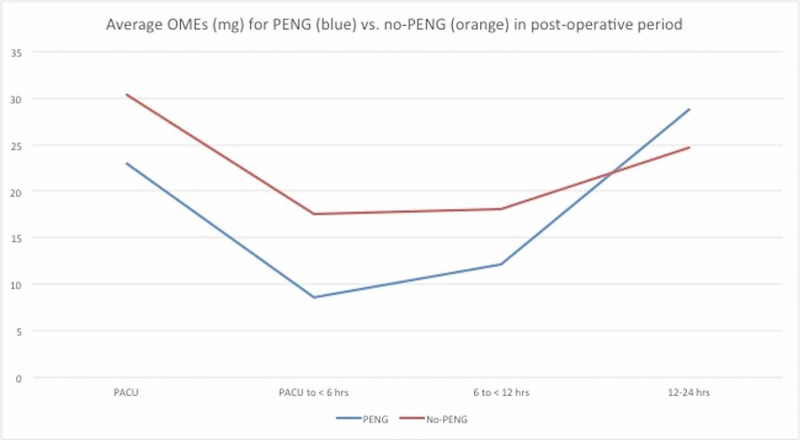
Average OMEs (mg) for PENG plus QL (blue) versus QL only (orange) groups for Revision THA. OME: Oral morphine equivalent; PENG: Pericapsular nerve group; QL: Quadratus lumborum; THA: Total hip arthroplasty.

Average opioid use in the QL group was higher in three of the four time periods when compared to the PENG plus QL group (Table 2, Figure 3).

**Table 2:** Post-operative opioid use of revision total hip arthroplasty patients after quadratus lumborum (QL) block versus pericapsular nerve group (PENG) plus QL block.

	QL N = 8	PENG + QL N = 8	Percentage change
Age (years)	59.6	59.8	
Post-operative OMEs			
PACU	30.4	23	↓ 24.3%
0 to < 6 hrs	17.5	8.6	↓ 50.9%
6 to < 12 hrs	18.1	12.2	↓ 32.6%
12 to 24 hrs	24.7	28.8	↑ 16.6%

The average oral morphine equivalents required in the first 12 hours after revision THA was 66.1 mg in the QL group and 43.8 mg in the PENG and QL group.

**Figure 4:**
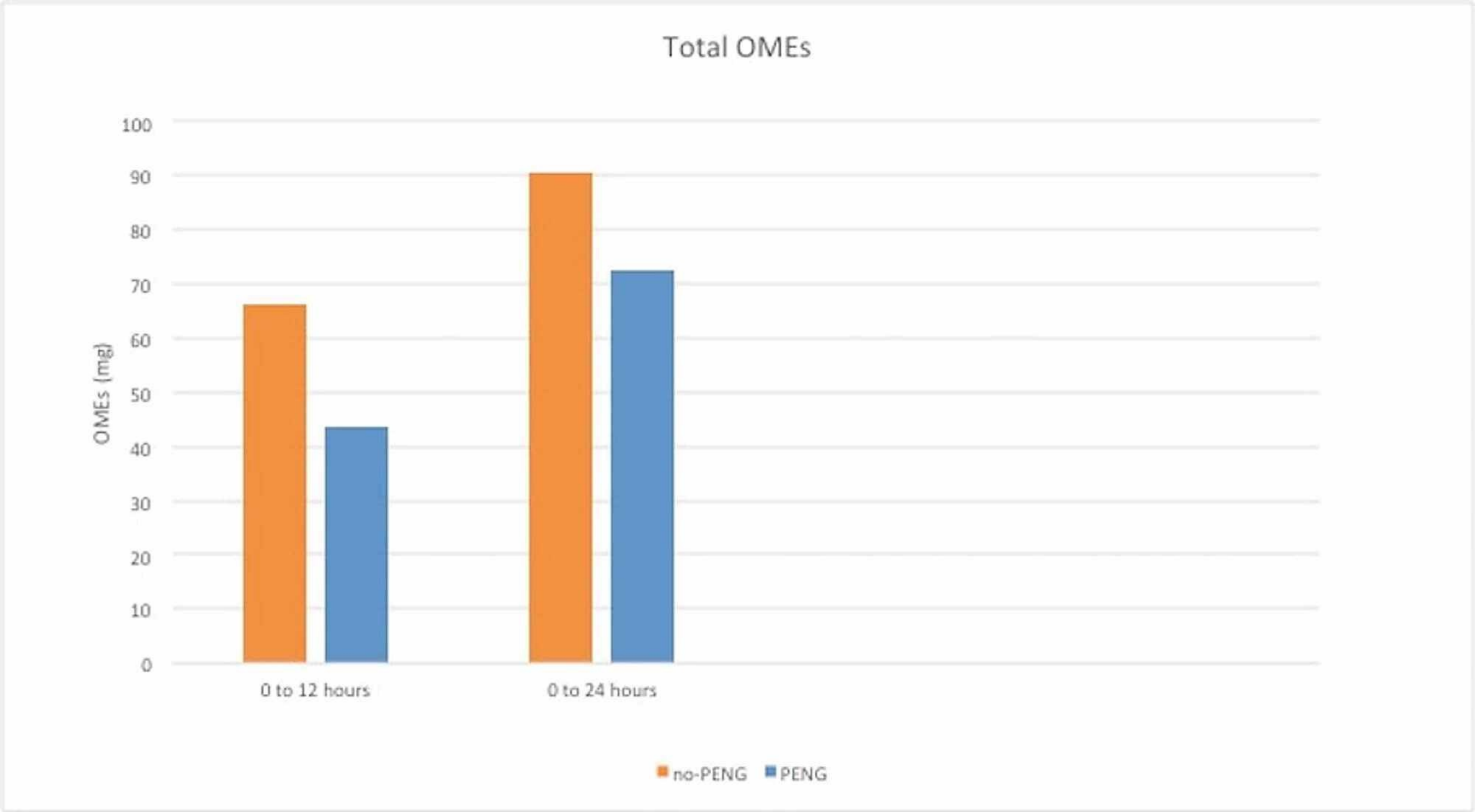
Total OMEs for QL only (orange) versus PENG plus QL (blue) groups in first 12 and first 24 hours in post-operative period. OME: Oral morphine equivalent; QL: Quadratus lumborum; PENG: Pericapsular nerve group.

The average oral morphine equivalents required in the first 24 hours after revision THA was 90.7 mg in the QL group and 72.6 mg in the PENG and QL group (Figure 4).

## Discussion

The innervation of hip joint is complex with anterior capsule supplied predominantly with nociceptive fibers and posterior capsule with mechanoreceptors [13]. The coverage of articular nerve supply to hip joint is critical for an effective analgesia. The PENG block is an interfascial plane block aiming to block the articular branches supplying anterior capsule to enable hip analgesia. A small case series published initially along with the description of this novel PENG block showed good analgesic benefit for hip fractures. The median reduction of pain scores in this study was 7 points, showing a larger decrease in pain scores compared to other regional techniques in hip fractures [3].

The benefits of the PENG block are patient positioning for procedure, no significant motor weakness (potential motor sparing effect) and analgesic efficacy [8]. The disadvantage is that it cannot be used as a sole anesthetic block for the hip surgery and it can be used in combination with other nerve blocks like FIB/FICB for more extensive analgesia for hip surgery. Recently, a randomized controlled study concluded that anterior quadratus lumborum provided effective analgesia and decreased opioid requirements up to 48 hours after primary THA [9]. In this case series, the PENG block was combined with the QL block to provide effective analgesia for revision THA. The PENG block covers branches of femoral nerve above inguinal ligament which innervates hip joint, and hence provides potential advantage in comparison to femoral nerve block.

In a recent case series utilizing PENG block for THA, postoperative pain scores and OME use were lower in the group undergoing primary hip surgery compared to revisions [1]. This reveals that PENG blocks may be a useful regional anesthetic technique for postoperative analgesia for primary hip surgery. The revision THA is more extensive surgery and may need effective coverage for both anterior and posterior capsule of hip joint besides surgical incision coverage. PENG blocks in combination with the quadratus lumborum (QL) block, or lateral femoral cutaneous (LFCN) nerve block or local infiltration analgesia (LIA) may be needed for revision THA.

To the best of our knowledge, this is the first retrospective case series study to combine the PENG block with the QL block for revision THA analgesia. The main findings of this study are superior analgesia with the combination blocks as compared to only QL block for revision THAs. Also opioid requirements in the combination group were lower up to at least the first 24 hours. Findings of the opioid sparing analgesic effect in the era of opioid epidemic and preservation of lower limb muscle strength in the era of accelerated physical therapy after THA are therefore encouraging.

The anterior lumbar QL block technique deposits local anesthetic deep to the anterior thoracolumbar fascia between the QL and psoas major muscles, in close proximity to the lumbar plexus with potential local anesthetic spread to the paravertebral (PVB) region. The anterior QL block aims to block the lumbar plexus branches, including the lateral femoral cutaneous nerve, which innervates the surgical incision site commonly used in THA approach. A recent cadaveric study and case series concluded that the supra-iliac approach to the anterior QL block involved T10-L3 dermatomal coverage and provided effective analgesia for total hip surgery [14].

Post-operative pain management after THA has always been a challenging goal to achieve. Multiple regional techniques have been used in the past, but there is not a “best proven intervention” for THA analgesia [5]. The main regional techniques for THA include lumbar plexus block, lumbar epidural, femoral nerve block, sciatic nerve block, fascia iliaca block (FIB), pericapsular injection or obturator nerve block. Unfortunately, all the above-mentioned blocks provide either inconsistent or partial analgesia, or are associated with lower extremity weakness that may interfere with physical therapy or increase the risk of fall. Use of a lumbar epidural catheter or inadvertent epidural spread of lumbar plexus block can result in hypotension, leg weakness and related adverse effects. The peripheral nerve blocks have been shown to be associated with falls after knee and hip arthroplasty [15]. The anterior approach for the QL block has also been shown to result in lower extremity weakness [12]. However, in this case series there was no incident of lower extremity weakness as supported in a recent prospective study [9].

Postoperative opioid requirements after THA vary depending on the type of regional anesthesia technique used. One study of continuous femoral nerve blocks for THA showed opioid requirement of approximately 160 mg of OMEs and another study of continuous lumbar plexus block showed approximately 114 mg OMEs in first 48 hours [16]. There are no prospective randomized studies where OMEs are compared among regional techniques specific for revision THA. In this retrospective case series the combination block group has lower OME total for first 12 hours (43.8 mg) and first 24 hours (72.6 mg) as compared to the QL only group for first 12 (66.1 mg) and 24 (90.7 mg) hours (Figure 4). There is an obvious trend of decreased opioid requirements, but has not reached statistical significance due to small sample size (Figure 3). There is slight increase in OMEs in the combination group during 12-24 hours after surgery which may explain better pain scores.

There are some limitations to this case series such as small sample size, retrospective design, and publication bias [17]. Also we did not check sensory dermatomal levels to verify effective coverage from the PENG block. The surgical approach for the THA may affect the severity of post-operative pain. Although in this study, all surgical approaches were posterolateral for revision THA. The complex innervation of hip and variation in anatomical planes where nerves run could explain the inconsistency of block results [18]. We did not study the effect of the regional blocks on ambulation and physical therapy in postoperative period.

At the same time, this case series explores a new combination approach for THA patients which can be utilized to provide effective analgesia for revision or complex hip arthroplasty. Large sample size studies are warranted to further understand this new technique and also to compare its efficacy with traditional blocks for hip analgesia. Also cadaveric and magnetic resonance imaging studies are required for better understanding of anatomical spread of local anesthetic and nerves covered with PENG block.

## Conclusions

The PENG plus anterior QL block provided adequate post-operative analgesia for patients undergoing revision THA. This combination block for revision THA reduced pain scores and had opioid sparing effects post operatively. The PENG block is an easy ultrasound-guided regional technique which can be performed in supine position for patient comfort. Further prospective randomized studies are warranted to determine the efficacy of PENG block for analgesia and quality of recovery after hip surgery. Also safety of PENG block alone or in combination with other blocks needs further investigation.
